# Are silica defences in grasses driving vole population cycles?

**DOI:** 10.1098/rsbl.2008.0106

**Published:** 2008-05-15

**Authors:** F.P Massey, M.J Smith, X Lambin, S.E Hartley

**Affiliations:** 1 Department of Biology and Environmental Science, University of SussexFalmer, Brighton BN1 9QG, UK; 2 School of Mathematical and Computer Sciences, Heriot-Watt UniversityEdinburgh EH14 4AS, UK; 3 Institute of Biological and Environmental Sciences, University of AberdeenTillydrone Avenue, Aberdeen AB24 2TZ, UK

**Keywords:** grass, herbivory, *Microtus*, population cycles, silica

## Abstract

Understanding the factors that drive species population dynamics is fundamental to biology. Cyclic populations of microtine rodents have been the most intensively studied to date, yet there remains great uncertainty over the mechanisms determining the dynamics of most of these populations. For one such population, we present preliminary evidence for a novel mechanism by which herbivore-induced reductions in plant quality alter herbivore life-history parameters and subsequent population growth. We tested the effect of high silica levels on the population growth and individual performance of voles (*Microtus agrestis*) reared on their winter food plant (*Deschampsia caespitosa*). In sites where the vole population density was high, silica levels in *D. caespitosa* leaves collected several months later were also high and vole populations subsequently declined; in sites where the vole densities were low, levels of silica were low and population density increased. High silica levels in their food reduced vole body mass by 0.5% a day. We argue that silica-based defences in grasses may play a key role in driving vole population cycles.

## 1. Introduction

One fundamental goal of ecology is to understand the temporal dynamics of populations. Cyclic populations are model systems for this purpose, showing clear and repeated population trajectories, despite stochasticity. It is now widely believed that trophic interactions are major determinants of cyclic dynamics, which are common in many herbivore populations including the classic example of grass-feeding voles. Vole cycles are often thought to result from their interactions with specialist predators (although see Graham & Lambin 2002); it is less commonly believed that cycles result from interactions with their food plants. Here, we present preliminary evidence to suggest a negative relationship between past vole abundance and the concentration of silica defences in their winter food plants. We hypothesize that this interaction could play a key role in generating the population cycles.

While cyclic vole populations occur in a wide range of ecosystems, in most instances, a grass-eating species (typically *Microtus* spp.) dominates the small mammal guild (but see Hanski & Henttonen 2002). Negative interactions with food plants need to occur with a sufficient time lag in order to induce population cycles. Grasses regrow after defoliation by mobilizing energy stored underground, but the resulting lag in regrowth is thought to be insufficiently slow to lead to cycles (Turchin & Batzli 2001). Grazing-induced reductions in plant quality may be important in some systems (Haukioja 1980), but grasses are thought to be low in induced chemical defences (Vicari & Bazely 1993). Previous studies have not identified any causal link between changes in food quality and vole population densities (Lindroth & Batzli 1986; Agrell *et al*. 1995; Klemola *et al*. 2000). Crucially, however, none of these studies has considered the role of silica, the principal defence in grasses (Massey *et al*. 2007*a*), which our previous work has found to reduce foliage digestibility severely for voles by restricting nitrogen absorption from grass leaves (Massey & Hartley 2006). It has long been known that past grazing leads to increases in future foliar silica levels, and to reductions in foliar palatability for mammals (McNaughton & Tarrants 1983; Gali-Muhtasib *et al*. 1992). Recently, we have demonstrated a slow induction response in grasses to repeated vole grazing, resulting in a fourfold increase in silica content (Massey *et al*. 2007*b*), to levels that affect vole performance (Massey & Hartley 2006). It is plausible that the build-up of sufficiently high vole densities could lead to population-limiting food shortages, through induced silica defences reducing food nutritional quality. If these nutritional deficiencies persist for sufficiently long after the vole population peak, they could lead to population cycles through a lagged negative feedback mechanism.

In this study, we test whether changes in the population density of the field vole *Microtus agrestis* L., and therefore grazing intensity, are related to changes in silica levels in *Deschampsia caespitosa* L. We selected this grass species as it is an important food plant to *M. agrestis* in northern Europe (Stenseth *et al*. 1977) and one of the few food plants available over winter. We also assess the impact of silica content of *D. caespitosa* leaves on the winter growth performance of female voles. Female body condition at the end of winter determines the onset of breeding in the following spring, a key predictor of future vole population growth (Smith *et al*. 2006).

## 2. Material and methods

### (a) Grass silica content and vole population density

We studied field voles in grassy clear-felled sites in Kielder Forest, northern England. Populations fluctuate cyclically between approximately 20 and 700 voles ha^−1^ in optimal habitats with a 3–5-year period (see Lambin *et al*. (2000) for details). Long-term monitoring shows that sites do not all fluctuate synchronously, making it possible to simultaneously sample sites at different stages of the population cycle (Bierman *et al*. 2006). Leaf samples of *D. caespitosa* were taken from four sites that had exhibited clear and contrasting population trajectories.

The samples of newly expanded leaves were taken from 10 randomly selected plants within each site. The leaves were dried at 80°C, ground using a ball mill (Pulversette 23, Fritsch) and analysed for silica in a scanning electron microscope (LEO 420 Stereoscan; Carl Zeiss) equipped with an energy-dispersive X-ray detector (Oxford Instruments EDX with INCA software). The samples were assessed for elemental composition for 5 min to identify and map silicon at two positions (Gong *et al*. 2006). An image analysis software (Adobe Photoshop v. 5.5, with image processing toolkit) was used to calculate the area of silicon as a proportion of the area of leaf material. The samples from SEM analysis were calibrated against 10 samples for which silicon was assessed using the colorimetric silicomolybdate technique (Allen 1989) and to convert values to percentage dry mass of SiO_2_ (linear calibration: *R*^2^=92%).

### (b) Vole growth performance

*Deschampsia caespitosa* was grown from seed in sward trays (20×30×5 cm) of peat : grit : perlite mix (3 : 2 : 1), under glasshouse conditions for 16 weeks (15–25°C, 12 L/12 D) and then moved to an unheated greenhouse one month prior to the vole performance study. Half of the plants were watered with 300 ml of silica solution (150 mg l^−1^ silica as NaSiO_3_·9H_2_O in dH_2_O). Ten samples were assessed for silica content using SEM (as above).

Ten adult female voles (20–24 g) were caught from a field site close to the University of Sussex in December 2006 and acclimatized to captivity for two weeks prior to the trials. Voles were held individually in glass tanks (45×30×30 cm, containing sawdust and cotton–wool bedding), in an unheated room next to open windows with no additional light (mean 7.7°C, range −0.7 to 15.5°C). They were randomly assigned to high- or low-silica diet treatments and given 15 g of fresh *D. caespitosa* leaves daily for 42 days (30 December 2006–13 February 2007), together with a standard dried diet (rabbit maintenance diet; B&K Feeds Universal) and water. The voles were weighed at weekly intervals.

## 3. Results

### (a) Grass silica content and vole population density

**Figure 1:**
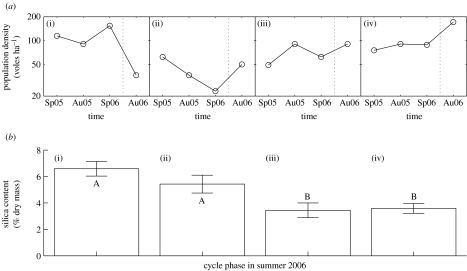
(*a*) Vole population densities from four sites at different phases of the population cycle from spring 2005 to autumn 2006 (Sp, spring; Au, autumn; 05/06, year 2005/2006) and (*b*) associated mean silica content of *D. caespitosa* leaves in summer 2006 (samples taken in October 2006) from each site (silica content: ANOVA *F*_3,36_=8.04, *p*<0.001, bars sharing common letters do not differ significantly). Note the log axis for the vole population density. (i) 1, Declining phase; (ii) 2, trough; (iii) 3, start of increase; (iv) 4, increasing phase.

Silica levels were highest in site 1, where the vole population density was high in the previous spring and declined over the study period (figure 1), and in site 2 where the vole population was at the trough in the population cycle. The silica content of *D. caespitosa* leaves was lowest at sites 3 and 4, where the vole population density was low in the previous spring and increased over the study period (figure 1). The silica levels of *D. caespitosa* in increasing sites were only 54% of the levels from the site where the population was in rapid decline.

### (b) Vole growth performance

**Figure 2:**
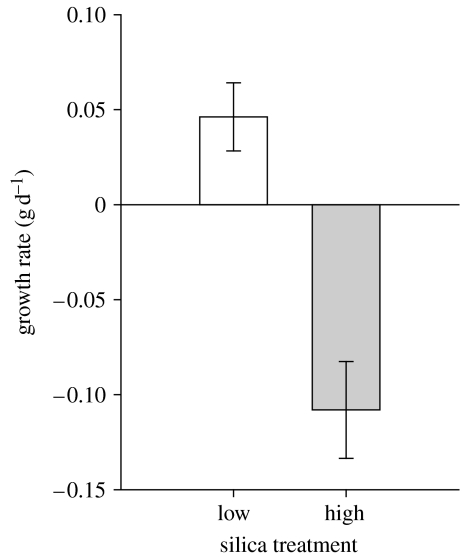
Winter growth rates of adult female voles (*n*=5) during December–January 2006 when reared on high- and low-silica diets of *D. caespitosa*. Values are means±s.e. (ANOVA: initial body mass (cov.) *F*_1,7_=7.70, *p*=0.027; silica *F*_1,7_=21.26, *p*=0.002).

The silica treatment significantly increased the foliar silica content of *D. caespitosa* leaves (low 1.79±0.09, high 6.62±0.10% dry mass, *t*-test: *t*=35.6, *p*<0.001, d.f.=17), at levels similar to those found at the field site. This increased silica content caused large reductions in vole growth rate, so that the voles reared on high-silica diets of *D. caespitosa* were losing 0.5% of body mass per day, while the voles on low-silica diets maintained a positive growth rate, gaining 0.25% of body mass per day (figure 2).

## 4. Discussion

This is the first study to link differences in the silica content of grasses, observed in the field, to the population densities of herbivores with cyclic dynamics. The silica content of *D. caespitosa* was higher where the vole populations were previously high but declining at the time of sampling, and low where they were increasing following a period of low density. We suggest that high population densities in the previous spring may have led to high levels of silica in the *D. caespitosa* leaves, and hence to a decline in vole densities by the following autumn. This is supported by recent field enclosure experiments that demonstrated a 45% increase in silica levels of *D. caespitosa* in response to high vole densities over a 12-month period (Smith 2008). Agrell *et al*. (1995) also found reduced growth rates and reproductive outputs in voles exposed to areas of previously high grazing intensity enclosures compared with voles in low grazing intensity enclosures with similar grass swards. We suggest silica may be the cause of such a difference.

This is the first demonstration of silica-induced reductions in growth using a dominant grass species of vole diets in northern Europe, and specifically on overwintering voles, the most significant period for determining future population growth. Reduced growth and vole body mass have consistently been observed in voles where populations are in decline, compared with increasing populations (Ergon *et al*. 2004). This study suggests that a cause for the decline may be the poor food quality of previously grazed grasses that reduces digestive efficiency in voles (Massey & Hartley 2006).

Our findings support the hypothesis that plant quality–vole abundance interactions could lead to population cycles. Periods of sustained grazing could cause silica induction in grasses, so that herbivores subsequently experience reduced availability of digestible nutrients. This in turn influences their body mass, growth rates and reproductive performance, most acutely over periods of energetic bottlenecks, i.e. the end of winter (Ergon *et al*. 2004). After periods of low grazing impact, silica induction in leaves is relaxed and less well-defended leaves are produced, such that herbivores are again able to access nutrients in grasses. This hypothesis is consistent with the results of a previous reciprocal field transplant experiment with the same cyclic field vole populations that pointed towards local environmental factors as the dominant determinants of cyclic life-history variation (Ergon *et al*. 2001).

The mechanism proposed here could plausibly occur in other plant–herbivore systems; for example, Hogstedt *et al*. (2005) identified four other grass-feeding herbivores with population dynamics similar to voles. Future empirical studies on herbivorous rodent population cycles should consider the role of grazing-induced plant quality changes, specifically silica in grasses, in driving population fluctuations.
